# Evidence quality and uncertainties considered in appraisal documents of drugs for rare diseases in England and Germany: a data extraction protocol

**DOI:** 10.1136/bmjopen-2024-089418

**Published:** 2025-02-16

**Authors:** Lea Wiedmann, Ellen Nolte, John Cairns

**Affiliations:** 1 Department of Health Services Research and Policy, London School of Hygiene & Tropical Medicine, London, UK

**Keywords:** Decision Making, Health economics, Health policy, Protocols & guidelines

## Abstract

**Introduction:**

Rare disease treatments (RDTs) promise considerable patient benefit but the evidence to demonstrate their value in health technology assessment (HTA) is often limited. HTA outcomes for RDTs vary across countries and there are differences in how uncertainty is dealt with by HTA agencies. Yet, there is limited comparative research assessing how different HTA agencies consider issues affecting evidence quality and uncertainty in RDT appraisals. This protocol describes a systematic and consistent approach for data extraction from RDT appraisal documents produced to inform decisions by HTA agencies. By documenting data extraction rules transparently, we ensure reproducibility and reliability of analyses of the extracted data.

**Methods and analysis:**

We will select RDT appraisals issued by the National Institute for Health and Care Excellence (NICE) in England and the Federal Joint Committee (GBA) in Germany, using predefined inclusion criteria. We will extract data from appraisal documents in accordance with the rules set out in this protocol. We will analyse the extracted data to investigate how issues affecting evidence quality and uncertainty as documented in appraisals are considered, highlighting the similarities and differences between countries and identifying factors that are associated with HTA outcomes.

**Ethics and dissemination:**

This study was approved by the Ethics Committee of the London School of Hygiene & Tropical Medicine (reference number 29156). Study results will be submitted for publication in peer-reviewed journals.

**STRENGTHS AND LIMITATIONS OF THIS STUDY**

- This protocol enables systematic, consistent and transparent extraction of data on evidence quality and uncertainties considered in appraisal documents of rare disease treatments (RDTs) produced by the National Institute for Health and Care Excellence (NICE) and the Federal Joint Committee (GBA)/Institute for Quality and Efficiency in Health Care (IQWiG) to inform health technology assessment (HTA) decisions.
- We assess key issues affecting evidence quality and uncertainty as recorded in appraisal documents, including characteristics of the HTA process, the RDTs, the clinical study evidence and economic models.
- This protocol is used to investigate RDT appraisals, and while research findings are specific to RDTs, the data extraction rules and analytical methods can also be applied to other (non-rare) treatments.
- This protocol is designed for data extraction from appraisal documents issued in England and Germany, but it may, with country-specific and process-specific modifications, also be applicable to HTA contexts in other settings.
- This protocol focuses on key issues affecting evidence quality and uncertainty documented in appraisals, rather than providing a full overview of all factors that influence evidence quality, or the methods used to explore uncertainty in HTA decision-making processes.

## Introduction

 Rare disease treatments (RDTs) have been associated with considerable patient benefit, especially for those conditions where there is no treatment available or for which new therapy delivers a substantial improvement over existing therapies.^1 2^ Some RDTs, particularly advanced therapy medicinal products (ATMPs), can be transformative, not only halting disease progression but also potentially curing or reversing disease processes.^3 4^ Yet, RDTs are typically high cost^5^,^9^ and the evidence demonstrating their clinical benefit is often limited^10 11^ because RDT trials tend to enrol fewer patients, are less likely to be randomised, are less likely to use active comparators and are more likely to be open label.^12^,^14^ As a consequence, clinical data on RDTs tend to be of lower quality, increasing uncertainty about the benefits to patients and challenging health technology assessment (HTA) agencies informing decision on whether to publicly fund RDTs.^15^,^19^

HTA agencies in different countries use different ways to manage uncertainty,^20^ with different methods for evaluating uncertainty in the clinical evidence^21^ and for identifying, exploring and communicating uncertainty in health economic modelling where this is used.^22^ Approaches used are highly context-dependent and shaped by perceptions of risk by individual agencies.^20 23^ Differences in approaches have been suggested as a potential reason for observed variation in HTA outcomes,^24^,^28^ but so far there is limited comparative evidence and detailed understanding of how HTA agencies deal with issues affecting evidence quality and uncertainty in RDT appraisals.

This protocol sets out an approach to systematically identify issues affecting evidence quality and uncertainties raised in RDT appraisal documents and to better understand variation in HTA decision-making in different settings. We focus on HTA agencies in two countries: the National Institute for Health and Care Excellence (NICE) whose positive recommendations are binding in England,^29 30^ and the Federal Joint Committee (GBA), the highest decision-making body in the German statutory system, which is supported by the Institute for Quality and Efficiency in Health Care (IQWiG) providing benefit assessments for regular appraisals in Germany.^31^ These countries were chosen because, first, they have well-established institutionalised processes with HTA agencies that provide independent and evidence-based reviews of health technologies and clear reimbursement procedures for treatments.^29 32^ Second, HTA systems in England and Germany likely differ in how they consider evidence quality and uncertainty as indicated by differences in HTA processes and outcomes^2629 33^,^36^ and within the wider context of how healthcare is governed, funded and organised.^37^ Third, both countries have introduced provisions that enable HTA agencies to issue a more favourable evaluation for some RDTs.^38^ For example, the NICE highly specialised technology (HST) appraisal guidance for RDTs that meet specific criteria posits a higher cost-effectiveness threshold.^30 39^ In Germany, new RDTs that have not exceeded a revenue threshold of €30 million per year are generally considered to have an added clinical benefit and therefore do not require comparison with an appropriate comparator therapy (ACT).^40^ Finally, in both countries, HTA reports are made publicly available and are accessible for research purposes.

The data extraction protocol presented in this paper seeks to support systematic, consistent and transparent data extraction from HTA appraisal documents for RDTs, thereby enabling a reproducible approach that minimises the risk of bias during data extraction. This protocol differs from previous work developing a methodological framework to identify reasons for diverging HTA outcomes^23^ in that it (1) focuses on specific issues affecting evidence quality and uncertainty in NICE and GBA appraisals, and (2) is designed to enable both qualitative and quantitative analyses of the extracted data, providing insights into similarities and differences between countries and identifying factors that are associated with HTA outcomes. Although the protocol focuses on HTA appraisal documents in England and Germany, we believe the approach presented here has wider applicability beyond these two countries. It allows, with some likely modification, for similar analyses to be conducted elsewhere, which will, in turn, enable systematic assessment of how issues affecting evidence quality and uncertainty are considered in RDT appraisal documents.

## Methods and analysis

We first present the approach for appraisal selection and the identification of medicine-indication pairs. We then outline categories used for data extraction (identification variables, RDT appraisal characteristics and the HTA outcome). Finally, we describe how we validate extracted data and describe options for data analysis. A table with abbreviations used in this paper is provided in the online supplemental material.

### Appraisal selection

We consider all completed RDT appraisals published by NICE and the GBA between 2011 and 2023 for inclusion in the selection according to predefined criteria (box 1). We chose this timeframe because in 2011, Germany introduced the early benefit assessment (EBA) process for the evaluation of new treatments, including RDTs.^41^

Box 1 **Appraisal selection criteria**

**Inclusion criteria**

- Completed appraisals for RDTs published between 1 January 2011 and 31 December 2023

**Exclusion criteria**

- Original appraisals if they have been replaced by updated guidance
- England:
  - Terminated appraisals
  - Withdrawn appraisals
  - Multiple technology appraisals
- Germany:
  - Terminated appraisals
  - Paused appraisals
  - Appraisals for RDTs that were exempted from benefit assessment

RDT=, rare disease treatment.

RDT appraisals in England will be identified using the Orphan Register of the Medicines & Healthcare products Regulatory Agency (MHRA)^42^ because the NICE guidance database does not allow filtering for RDTs. Where an appraisal published in the Technology Appraisal (TA) guidance^43^ was available for a treatment listed as a rare disease product in the MHRA Orphan Register, we consider this to be an RDT appraisal. In addition, we consider all appraisals published in the HST appraisal guidance for inclusion.^44^ For Germany, we will use the publicly available online appraisal database of the GBA, applying the rare disease filter to identify RDTs.^45^

### Data sources

Published appraisal documents are the primary data source for the analysis. We consider the following appraisal documents issued by NICE: (1) the final scope, (2) committee papers, (3) the final appraisal determination (FAD) or final evaluation determination (FED) document, and (4) public committee presentations. For Germany, we consider the following appraisal documents: (1) the evidence submission by the manufacturer (*Dossier*), (2) the benefit assessment (*Nutzenbewertung*) either conducted by IQWiG or the GBA, (3) the justification document (*Tragende Gründe*) and (4) the appraisal resolution (*Beschlusstext*).

### Indication pairs

From the selected appraisals, we will focus on indications for which a HTA outcome is available in both settings. We treat these indications as ‘medicine-indication pairs’. Medicine-indication pairs can be either a ‘matched indication pair’, which means that the HTA outcome applies to exactly the same therapeutic indication for a RDT in both countries, or a ‘partially matched indication pair’, namely, the approved therapeutic indication for the appraised RDT is the same in both countries, but the eligible population differs. For example, metreleptin for the treatment of partial lipodystrophy (a condition in which the body is unable to produce and maintain healthy fat tissue) among patients aged 12 years and older for whom standard treatments have not achieved adequate metabolic control was assigned a non-quantifiable added clinical benefit in Germany and is therefore available to all patients with this condition.^46^ Conversely, in England, the RDT was only recommended for patients who meet certain criteria (ie, blood sugar HbA1c level above 58 mmol/mol (7.5%), and/or fasting triglycerides above 5.0 mmol/L).^47^

#### HTA outcomes for subgroups

Our preliminary assessment of appraisal documents found that, in Germany, appraisals for some RDTs differentiate between indication subgroups. For example, with regard to nusinersen for spinal muscular atrophy, the GBA issued separate clinical benefit ratings (CBRs) for three different subgroups of presymptomatic patients with different genetic profiles with regard to the survival motor neuron (SMN) 2 gene,^48^ while the NICE appraisal for nusinersen did not distinguish between these subgroups.^49^ In such cases, we used the GBA appraisal that included the largest subgroup to arrive at a (partially) matched-indication pair.

#### Consideration of appraisal timelines

Appraisals undertaken by NICE and GBA follow different HTA process timelines after the approval of a given treatment. For example, caplacizumab for the treatment of acquired thrombotic thrombocytopenic purpura (aTTP), a rare disorder of the blood coagulation system, first obtained marketing authorisation by the European Medicines Agency (EMA) in 2018 and the licence was restricted for the treatment of adults only.^50^ The GBA issued a CBR for this indication in the same year.^51^ Because the approved indication was extended by the EMA in 2020 to also include adolescents (≥ 12 years),^52^ the GBA issued an additional CBR for this patient group.^53^ Conversely, NICE published only one appraisal, in 2020, and this recommended the use of caplacizumab for aTTP for all people aged 12 years and older.^54^ Where indications differed due to diverging appraisal timelines, we chose the indication covering the largest population; in the case of caplacizumab, we would select the 2018 GBA CBR which covered all adults with aTTP.

### Data extraction

**Figure 1:**
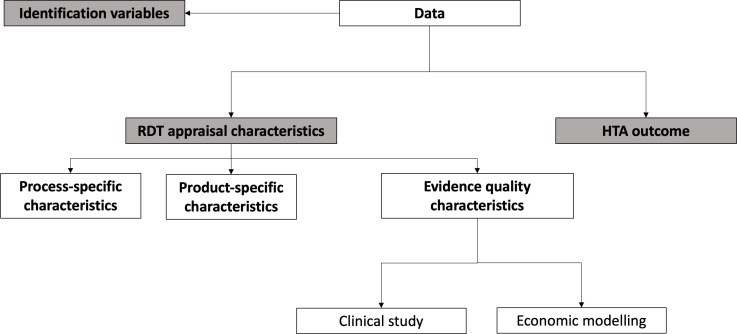
Data extraction schema. HTA, health technology assessment; RDT, rare disease treatment.

Figure 1 shows the three main categories that we will use for data extraction: identification variables, RDT appraisal characteristics and the HTA outcome. We explain each category in turn.

#### Identification variables

Identification variables refer to variables that provide background information of each medicine-indication pair. For this study, the following identification variables will be collected: identification number of the appraisal; the brand name and active substance; indication; and the appraisal start and publication date.

#### HTA outcome

Our main dependent variable is the HTA outcome. The HTA outcome in England directly informs decision-making on whether a treatment is adopted and thus publicly funded, and in Germany, it informs subsequent drug price negotiations. For Germany, we will record the CBR (major, considerable, minor, non-quantifiable added benefit, no added benefit, benefit lower than the ACT). For England, we will record whether an RDT was recommended for routine commissioning and whether it was recommended in line with marketing authorisation. We will use the extracted data to better understand HTA outcomes in each country and how they differ.

Because HTA outcomes differ, it is important to consider possible approaches for a comparative analysis. For example, each indication pair could be placed into one of the following three categories: positive decision (‘List’), positive restricted decision (‘List with criteria (LWC)’) and no positive decision (‘Do not list (DNL)’) similar to the approach used by Kanavos *et al*.^55^ We will review the HTA outcomes recorded in each country after data extraction has been completed to inform our analytical approach.

#### RDT appraisal characteristics

We define process-specific characteristics, product-specific characteristics and evidence quality characteristics based on previous publications assessing drivers of HTA outcomes across settings.^2528 31 55^,^58^ The selection of characteristics was also informed, in part, by differences in HTA approaches used in England and Germany (see below).

Process-specific characteristics describe the HTA process in each country and include the guidance type (HST or TA guidance in England; regular or limited appraisals in Germany), whether an appraisal was a re-appraisal, and the reason for appraisal in Germany. Product-specific characteristics describe the characteristics of the treatment itself, for example, whether the RDT was classified as ATMP; whether the RDT was indicated for children, adults or both; the therapeutic area of the RDT; and whether an alternative active treatment for the indication was available.

Evidence quality variables include (1) clinical study variables and (2) economic modelling variables. Clinical study variables relate to key characteristics of the main study, such as the study design, the type of comparison conducted, the extent of the maturity of survival data, the risk of bias and the applicability of study results to clinical practice. Economic modelling variables relate to the model type used and uncertainties in the economic modelling evidence as described by the NICE committee in the FAD/FED. Because the final economic modelling approach accepted by the NICE committee can differ substantially from the original economic model submitted by the manufacturer, extracting data from the FAD/FED enables us to capture important challenges and uncertainties that are likely to influence the final reimbursement decision.

A provisional coding manual for this study can be obtained upon request from the authors.

#### Further country-specific considerations

##### Type of HTA performed

HTA approaches in England and Germany differ in one main dimension: NICE uses clinical and cost-effectiveness analyses^30^ and evaluations are based on a value-based assessment using a cost per quality-adjusted life year (QALY) threshold. NICE appraisals also consider other elements of value, for example, disease severity or treatment innovativeness.^29^ Conversely, the GBA typically does not use the cost per QALY metric.^35^ Instead, evaluations use a comparative clinical benefit assessment. New health technologies (or new indications) must undergo the EBA process,^59^ which determines a CBR. The CBR then forms the basis for the reimbursement price of the health technology, which is negotiated between the National Association of Statutory Health Insurance Funds and the manufacturer.^60^

Differences in HTA approaches mean that the clinical evidence submitted by the manufacturer is considered differently by NICE and the GBA. Thus, NICE typically reviews all clinical studies submitted by the manufacturer and notes whether data from clinical trials were used to inform the economic model. Conversely, the GBA may decide to not consider a given study that was submitted by the manufacturer to derive the CBR. For example, unadjusted indirect comparisons are usually not accepted.^61^,^63^ These differences are important and will be recorded during data extraction. Specifically, we will note whether the GBA accepted/rejected clinical studies, including reasons for non-acceptance, and whether NICE used clinical studies to inform the economic model in their appraisal.

##### Role of the licenced indication

In both countries, the remit of the appraisal is typically the indication for which a technology has received marketing authorisation (or licence). However, there may be differences between the administration of the intervention or comparator, or the patients included in the submitted clinical studies and the approved licence. Where this is the case, NICE often restricts the eligible population for which a drug is recommended and issues an optimised recommendation. For example, in TA589 (blinatumomab for acute lymphoblastic leukaemia in remission with minimal residual disease activity), NICE only recommended the RDT for adults with disease in first complete remission because the manufacturer did not present evidence for adults in second remission even though these patients are also covered by the licence.^64^ Similarly, in TA478 (brentuximab vedotin for relapsed or refractory systemic anaplastic large cell lymphoma), NICE only recommended the RDT for patients with an Eastern Cooperative Oncology Group performance status of 0 or 1 reflecting the patient population in the trial evidence even though the licence does not specify performance status.^65^ In contrast, and as noted earlier, in Germany, new treatments are usually recommended in line with the marketing authorisation, even if the available evidence does not include all patients covered by it. Taking the example of brentuximab vedotin for the treatment of CD30-positive cutaneous T cell lymphoma, the main trial considered in the appraisal excluded more aggressive and fatal subtypes of the disease; however, the GBA issued a minor added CBR for the whole population covered by the licence.^66^ For data extraction purposes, we record any statements made by the HTA bodies about the extent to which the submitted clinical evidence covers the population of the licence.

##### Redacted information

Redaction of data, particularly from clinical studies, is common in NICE appraisal documents^67^ and presents a challenge for extracting and analysing data. Where data is redacted, we will check whether the required information is available in appraisal documents issued in Germany for the same indication pair. For example, if the same data cut from a clinical study are used in the appraisal for the same indication in both countries but clinical data in NICE appraisals are redacted, we will extract the information from German appraisal documents. Alternatively, we will seek to identify the original publication of the study and extract related data accordingly.

##### Level of aggregation

Another important consideration is the level of aggregation of the extracted data. For example, sometimes several clinical studies are submitted to demonstrate the evidence for an RDT in an appraisal. All studies submitted as part of the appraisal will be recorded in both countries. However, to facilitate analysis of the extracted data, we will define a main study for each appraisal and use it for comparison between countries.

In addition, for the purpose of this study, economic uncertainties as identified by the NICE committee in the FAD/FED will be recorded. This represents a more aggregated level of information than the descriptions provided in the evidence assessment group (EAG) report; as such, it is likely that some issues that were discussed by the EAG but that were not included in the FAD/FAD might not be recorded. However, our focus on the FAD/FED aims to capture important uncertainties that likely affect decision-making while keeping data collection and analysis manageable and meaningful.

### Validation

This protocol will be validated independently by two external researchers who will repeat the data extraction for a random sample of indication pairs (approximately 15%). Researchers will compare extracted data and resolve disagreements by discussion to improve clarity and reduce subjectivity.

### Analysis

Extracted data will be analysed in MS Excel 2024 and R.^68^ We will present descriptive statistics, including proportions and cross-tabulations, for all collected characteristics. We will use Cohen’s kappa score to determine the level of agreement for some characteristics between both settings. We will compare HTA outcomes, discuss similarities and differences in approaches taken and explore reasons for potential differences. We will also explore which factors are associated with HTA outcomes. Following a preliminary assessment of appraisals, we have formulated working hypotheses about positive HTA outcomes (box 2).

Box 2 **Working hypotheses**

Product-specific characteristics

1. Appraisals for ATMPs are associated with a positive HTA outcome.
2. Appraisals for oncology RDTs are associated with a positive HTA outcome.
3. Appraisals for RDTs indicated for adults and children and children only are associated with a positive HTA outcome.
4. Appraisals for which no alternative active treatment was available are associated with a positive HTA outcome.

Clinical evidence quality characteristics

1. Appraisals in which the main study was an RCT are associated with a positive HTA outcome.
2. Appraisals in which the risk of bias in the main study was low are associated with a positive HTA outcome.
3. Appraisals in which the applicability of the main study was acceptable are associated with a positive HTA outcome.
4. Appraisals in which survival data was mature are associated with a positive HTA outcome.

ATMP=, advanced therapy medicinal product; HTA=, health technology assessment; RCT=, randomised control trial; RDT=, rare disease treatment.

### Patient and public involvement

None.

## Ethics and dissemination

This study was approved by the Ethics Committee of the London School of Hygiene & Tropical Medicine (reference number 29156). Data extraction is currently in progress, with completion of data extraction and analysis anticipated by the end of 2025. Results of the study will be submitted for publication in peer-reviewed journals.

## Discussion

This protocol provides an important addition to the literature that has sought to systematically analyse and understand differences in HTA outcomes across countries. We describe rules to extract data from appraisal documents issued in England and Germany, thereby allowing for comparative and replicable analyses. It provides a clear and transparent record of how data will be extracted and so ensure consistency in data extraction and minimisation of errors and biases. By documenting the data extraction approach for both countries in a transparent way, we ensure reproducibility and strengthen the reliability of the findings of analyses of the extracted data. In addition, our study findings may be used to inform methods of evidence interpretation in RDT appraisals in England and Germany and highlight similarities and differences of the approaches followed in both countries. More generally, the results of this study can be used as a baseline for comparative analyses of HTA processes and outcomes following the start of joint clinical assessments for RDTs as part of the EU HTA regulation in 2028.^69 70^

This protocol has some limitations. First, it is used to investigate RDT appraisals, which means that the research findings are likely to be RDT-specific. However, we believe that the extraction rules and analytical methods proposed may provide a helpful guide for the analysis for appraisals of other (non-rare) treatments. Second, the protocol has been specifically designed for a comparative analysis of appraisal documents issued in England and Germany. As such, it may not be fully applicable to HTA processes in other countries. However, with appropriate country-specific and process-specific modifications, we believe that our approach can helpfully inform similar analyses of the use of clinical and economic evidence in HTA processes in other countries that employ comparative clinical effectiveness or cost-effectiveness approaches. In this context, one area for further research would be to investigate our hypotheses (box 2) in other settings. Third, this protocol focuses on key issues affecting evidence quality and uncertainty in HTA processes, including the design of clinical studies, the type of comparison made, the risk of bias, applicability, the maturity of survival data, and economic modelling uncertainties. It does not provide a full assessment of all factors that influence evidence quality, or the methods used to explore uncertainty in HTA decision-making processes.

## supplementary material

10.1136/bmjopen-2024-089418 online supplemental file 1
