# Supplementary material for: Evidence quality and uncertainties considered in appraisal documents of drugs for rare diseases in England and Germany: a data extraction protocol
Source: BMJ Open. 2025 Feb 16;15(2):e089418. doi: 10.1136/bmjopen-2024-089418 (PMC11831311; doi:10.1136/bmjopen-2024-089418)
Supplement: online supplemental file 1 [file bmjopen-15-2-s001.docx]

Evidence quality and uncertainties considered in appraisal documents of drugs for rare diseases in England and Germany: A data extraction protocol

Supplementary material

Contents

[Supplementary material 1. List of abbreviations 2](#_Toc187230740)

# Supplementary material 1. List of abbreviations

Table 1: List of abbreviations

| aTTP | acquired thrombotic thrombocytopenic purpura |
| --- | --- |
| ACT | Appropriate comparator therapy |
| ATMP | Advanced therapy medicinal product |
| CBR | Clinical benefit rating |
| DNL | Do not list |
| EAG | Evidence Assessment Group |
| ECOG | Eastern Cooperative Oncology Group |
| EBA | Early benefit assessment |
| EMA | European Medicines Agency |
| FAD | Final appraisal determination document |
| FED | Final appraisal evaluation document |
| GBA | Gemeinsamer Bundesausschuss [Federal Joint Committee] |
| HTA | Health Technology Assessment |
| HST | Highly specialised technology guidance |
| IQWiG | Institut für Qualität und Wirtschaftlichkeit im Gesundheitswesen [Institute for Quality and Efficiency in Health Care] |
| L | List |
| LWC | List with criteria |
| MHRA | Medicines & Healthcare products Regulatory Agency |
| NICE | National Institute for Health and Care Excellence |
| QALY | Quality-adjusted life year |
| QoL | Quality of life |
| RDT | Rare disease treatment |
| SMA | Spinal muscular atrophy |
| SMN | Survival motor neuron |
| TA | Technology appraisal guidance |
