# Supplementary material for: Evidence quality and uncertainties considered in appraisal documents of drugs for rare diseases in England and Germany: a data extraction protocol
Source: BMJ Open. 2025 Feb 16;15(2):e089418. doi: 10.1136/bmjopen-2024-089418 (PMC11831311; doi:10.1136/bmjopen-2024-089418)
Supplement: Supplementary file 2 [file bmjopen-2024-089418.reviewer_comments.pdf]

PEER REVIEW HISTORY

BMJ Open publishes all reviews undertaken for accepted manuscripts. Reviewers are asked to complete a checklist review form and are provided with free text boxes to elaborate on their assessment. These free text comments are reproduced below.

ARTICLE DETAILS

Title (Provisional)

Evidence quality and uncertainties considered in appraisal documents of drugs for rare diseases in England and Germany: a data extraction protocol

Authors

Wiedmann, Lea; Nolte, Ellen; Cairns, John

VERSION 1 - REVIEW

**Reviewer**	**1**
**Name**	**Gozzo, Lucia**
**Affiliation**	**University of Catania**
**Date**	**12-Sep-2024**
**COI**	**None**

The protocol is well designed and will inform about the way the Regulatory Agency face the uncertainty about evidence for treatments for rare diseases.

However it should be useful to better describe the framework, including previous studies on the topic (Blonda A et al. Assessing the Value of Nusinersen for Spinal Muscular Atrophy: A Comparative Analysis of Reimbursement Submission and Appraisal in European Countries. Front Pharmacol. 2022 Jan 21;12:750742. doi: 10.3389/fphar.2021.750742; Stafinski T et al. HTA decision-making for drugs for rare diseases: comparison of processes across countries. Orphanet J Rare Dis. 2022 Jul 8;17(1):258. doi: 10.1186/s13023-022-02397-4; Blonda A et al. How Can We Optimize the Value Assessment and Appraisal of Orphan Drugs for Reimbursement Purposes? A Qualitative Interview Study Across European Countries. Front Pharmacol. 2022 Jul 19;13:902150. doi: 10.3389/fphar.2022.902150; Sarri G, Rizzo M, Upadhyaya S, Paly VF, Hernandez L. Navigating the unknown: how to best 'reflect' standard of care in indications without a dedicated treatment pathway in health technology assessment submissions. J Comp Eff Res. 2024 Feb;13(2):e230145. doi: 10.57264/cer-2023-0145; Gozzo L, Romano GL, Romano F, Brancati S, Longo L, Vitale DC, Drago F. Health Technology Assessment of Advanced Therapy Medicinal Products: Comparison Among 3 European Countries. Front Pharmacol. 2021 Oct 8;12:755052. doi: 10.3389/fphar.2021.755052; Dayer VW, Drummond MF, Dabbous O, Toumi M, Neumann P,

Tunis S, Teich N, Saleh S, Persson U, von der Schulenburg JG, Malone DC, Salimullah T, Sullivan SD. Real-world evidence for coverage determination of treatments for rare diseases. Orphanet J Rare Dis. 2024 Feb 7;19(1):47. doi: 10.1186/s13023-024-03041-z; Zisis K, Pavi E, Geitona M, Athanasakis K. Real-world data: a comprehensive literature review on the barriers, challenges, and opportunities associated with their inclusion in the health technology assessment process. J Pharm Pharm Sci. 2024 Feb 28;27:12302. doi: 10.3389/jpps.2024.12302; Jakubowski S, Kawalec P, Holko P, Kowalska-Bobko I, Kamusheva M, Petrova G, Draganić P, Fuksa L, Männik A, Ispán F, Briedis V, Bianchi I, Paveliu MS, Tesar T. Clinical aspects of reimbursement policies for orphan drugs in Central and Eastern European countries. Front Pharmacol. 2024 Mar 8;15:1369178. doi: 10.3389/fphar.2024.1369178; Macabeo B, Quenéchdu A, Aballéa S, François C, Boyer L, Laramée P. Methods for Indirect Treatment Comparison: Results from a Systematic Literature Review. J Mark Access Health Policy. 2024 Apr 16;12(2):58-80. doi: 10.3390/jmahp12020006; Castanon A, Sloan R, Arocha LS, Ramagopalan SV. EU HTA Joint Clinical Assessment: are patients with rare disease going to lose out? J Comp Eff Res. 2024 Jun;13(6):e240052. doi: 10.57264/cer-2024-0052; Felippini A, Biglia LV, Lima TM, Aguiar PM. HTA criteria adopted in different models of public healthcare systems for orphan drugs: A scoping review. Health Policy. 2024 Jun;144:105080. doi: 10.1016/j.healthpol.2024.105080), and the Regulation on health technology assessment.

**Reviewer**	**2**
**Name**	**Jonker, Anneliene Hechtelt**
**Affiliation**	**Univ Twente**
**Date**	**07-Jan-2025**
**COI**	**I declare that I have no competing interests**

Dear authors,

Thank you for this clear protocol.

as this is a protocol, I would only like to make minor suggestions. Firstly. it would be really helpful to have a list of abbreviations included. Secondly, for the identification variables, is anything included for the prevalence of the disease? Prevalence might have a big influence on the data quality that is available? For dependent variables, are also other therapy types recorded, in addition to ATMP, such as biologics, or small molecules?

VERSION 1 - AUTHOR RESPONSE

No.	Editor/Reviewer Comment	Author response
**Reviewer 1 Comments**		

6	Comments to the Author:<br>The protocol is well designed and will inform about the way the Regulatory Agency face the uncertainty about evidence for treatments for rare diseases.<br><br>However it should be useful to better describe the framework, including previous studies on the topic<br><br>• Blonda A et al. (2022a). doi: 10.3389/fphar.2021.750742<br>• Stafinski T et al. (2022). doi: 10.1186/s13023-022-02397-4<br>• Blonda A et al. (2022b). doi: 10.3389/fphar.2022.90215<br>• Sarri G et al. doi: 10.57264/cer-2023-0145<br>• Gozzo L et al. (2021). doi: 10.3389/fphar.2021.75505<br>• Dayer VW et al. (2024). doi: 10.1186/s13023-024-03041-z<br>• Zisis K et al. (2024). doi: 10.3389/jpps.2024.12302<br>• Jakubowski S et al. (2024). doi: 10.3389/fphar.2024.1369178<br>• Macabeo B et al. (2024). doi: 10.3390/jmahp12020006<br>• Castanon A et al. (2024). doi: 10.57264/cer-2024-0052<br>• Felippini A et al. (2024). doi: 10.1016/j.healthpol.2024.105080)	Thank you for your comment.<br><br>We have already referred to the publication by Stafinski et al. in our manuscript. Following your comment, we have now also included some references to previous studies on the topic (Blonda A et al. 2022a; Gozzo et al.; Felippini et al.).<br><br>We have not included some of the suggested references as they do not directly relate to comparative studies including England and Germany (Jakubowski et al; Macabeo et al.), or because they focus on investigating the use of real-world data which this protocol was not primarily designed to investigate (Dayer et al.; Zisis et al.).<br><br>We have also amended the discussion section to include references to the EU HTA regulation as follows:<br><br>More generally, the findings of this study can be used as a baseline for comparative studies of HTA processes and outcomes following the start of joint clinical assessments (JCA) for RDTs as part of the EU HTA regulation in 2028 (2, 3).
**Reviewer 2 Comments**		
7	Firstly. it would be really helpful to have a list of abbreviations included.	We agree with your comment. We have now added a list of abbreviations in the supplementary material.
8	Secondly, for the identification variables, is anything included for the prevalence of the disease? Prevalence might have a big influence on the data quality that is available? For dependent variables, are also other therapy types recorded, in addition to ATMP, such as biologics, or small molecules?	Thank you for your comment.<br><br>All RDTs in our data set fall within the prevalence definition of orphan designation by the European Commission. Providing reliable prevalence estimates for the indication corresponding for each HTA outcome in our data set is difficult because (i) recommendations by NICE are often restricted to a subgroup of the licensed population (optimised recommendation) for which prevalence estimates are not routinely reported and (ii) similarly, in HTA documents published by the GBA, prevalence estimates

		are typically not split by subgroups for which different clinical benefit ratings are issued.<br><br>Given that we expect a small data set, introducing additional dependent variables in relation to therapy type would increase uncertainty around the estimated effect in the subsequent quantitative analysis. We also did not specify hypotheses about the association between biologics or small molecules and the HTA outcome, so we prefer to focus on our main hypotheses.

References

1. Welsh Government. National Institute for Health and Care Excellence (NICE) guidelines: Guidance for health boards, trusts and other stakeholders on how to follow NICE guidelines. 11 September 2024. Available from: https://www.gov.wales/national-institute-health-and-care-excellence-nice-guidelines?utm_source=chatgpt.com (accessed 8 January, 2025).
2. Walker J. 22 October 2024. European Commission progresses EU HTA Regulation with second adopted implementing act [Internet]. [cited 8 January 2025]. Available from: https://becarispublishing.com/digital-content/blog-post/european-commission-progresses-eu-hta-regulation-second-adopted-implementing-act.
3. European Commission (EC). Implementation of the Regulation on health technology assessment 2025. Available from: https://health.ec.europa.eu/health-technology-assessment/implementation-regulation-health-technology-assessment_en (accessed 8 January, 2025).
